# Ethyl 5-[(1*H*-benzoimidazol-2-yl)amino­carbon­yl]-4-hydr­oxy-2-methyl-6-oxo-1-propyl-1,6-dihydro­pyridine-3-carboxyl­ate–ethanol–methanol (4/2/1)

**DOI:** 10.1107/S1600536809026816

**Published:** 2009-07-25

**Authors:** Svetlana V. Shishkina, Oleg V. Shishkin, Igor V. Ukrainets, Andrei A. Tkach, Lina A. Grinevich

**Affiliations:** a STC "Institute for Single Crystals", National Academy of Sciences of Ukraine, 60 Lenina ave., Kharkiv 61001, Ukraine; b National University of Pharmacy, 4 Blyukhera ave., Kharkiv 61002, Ukraine

## Abstract

The asymmetric unit of the title compound, 4C_20_H_22_N_4_O_5_·2C_2_H_6_O·CH_4_O, contains two pyridine-3-carboxyl­ate mol­ecules, one ethanol mol­ecule and one methanol mol­ecule disordered about in a centre of symmetry. The pyridinone ring, the carbamide group and the bicyclic fragment in both independent mol­ecules are planar within 0.03 Å due to the formation of intra­molecular O—H⋯O and N—H⋯O hydrogen bonds. The formation of these latter inter­actions also causes the redistribution of the electron density within the hydroxy­pyridone fragment, with the result that some bonds are elongated compared with values in the literature and some others are shorter. In the crystal phase, the pyridine-3-carboxyl­ate mol­ecules form layers parallel to (010), which are inter­linked through hydrogen bonds mediated by the bridging solvate mol­ecules. A terminal ethyl group in one of the mol­ecules is disordered over two sites of equally occupancy.

## Related literature

For general properties of *N*-acylic derivatives of 2-amino­benzoimidazole, see: Ukrainets *et al.* (1993 ▶, 2006 ▶). For the geometrical properties of related compounds, see: Bürgi & Dunitz (1994 ▶); Low & Wilson (1983 ▶). 
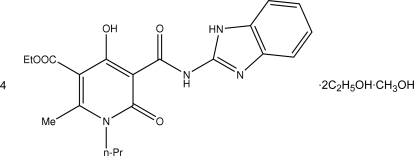


## Experimental

### 

#### Crystal data

4C_20_H_22_N_4_O_5_·2C_2_H_6_O·CH_4_O
*M* *_r_* = 1717.84
Triclinic, 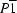

*a* = 10.5527 (4) Å
*b* = 13.9720 (4) Å
*c* = 16.0242 (5) Å
α = 86.804 (3)°
β = 70.980 (3)°
γ = 78.652 (3)°
*V* = 2189.90 (12) Å^3^
*Z* = 1
Mo *K*α radiation
μ = 0.10 mm^−1^
*T* = 293 K
0.40 × 0.30 × 0.10 mm

#### Data collection

Oxford Diffraction Xcalibur3 diffractometer
Absorption correction: none
23866 measured reflections
7499 independent reflections
3785 reflections with *I* > 2σ(*I*)
*R*_int_ = 0.030

#### Refinement

*R*[*F* ^2^ > 2σ(*F* ^2^)] = 0.055
*wR*(*F* ^2^) = 0.170
*S* = 0.89
7499 reflections
596 parameters
9 restraints
H-atom parameters constrained
Δρ_max_ = 0.44 e Å^−3^
Δρ_min_ = −0.35 e Å^−3^

### 

Data collection: *CrysAlis CCD* (Oxford Diffraction, 2005 ▶); cell refinement: *CrysAlis RED* (Oxford Diffraction, 2005 ▶); data reduction: *CrysAlis RED*; program(s) used to solve structure: *SHELXTL* (Sheldrick, 2008 ▶); program(s) used to refine structure: *SHELXTL*; molecular graphics: *XP* (Bruker, 1998 ▶); software used to prepare material for publication: *SHELXTL*.

## Supplementary Material

Crystal structure: contains datablocks I, global. DOI: 10.1107/S1600536809026816/bg2271sup1.cif

Structure factors: contains datablocks I. DOI: 10.1107/S1600536809026816/bg2271Isup2.hkl

Additional supplementary materials:  crystallographic information; 3D view; checkCIF report

## Figures and Tables

**Table 1:** Hydrogen-bond geometry (Å, °)

*D*—H⋯*A*	*D*—H	H⋯*A*	*D*⋯*A*	*D*—H⋯*A*
O1*A*—H1*A*⋯O3*A*	0.82	1.77	2.503 (2)	149
O1*B*—H1*B*⋯O3*B*	0.82	1.78	2.508 (2)	148
N2*A*—H2*A*⋯O2*A*	0.86	1.88	2.600 (3)	140
N2*B*—H2*B*⋯O2*B*	0.86	1.88	2.596 (3)	139
N3*A*—H3*A*⋯O2^i^	0.86	2.34	2.968 (3)	130
N3*A*—H3*A*⋯O3*A*	0.86	2.21	2.710 (3)	117
N3*B*—H3*B*⋯O1^ii^	0.86	2.46	3.106 (6)	132
N3*B*—H3*B*⋯O3*B*	0.86	2.18	2.691 (3)	118
O2—H2⋯N4*A*	0.82	2.27	2.926 (3)	138
O1—H1⋯N4*B*	0.82	2.35	3.050 (6)	144

Symmetry codes: (i) 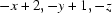; (ii) 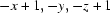.

**Fig. 1.**
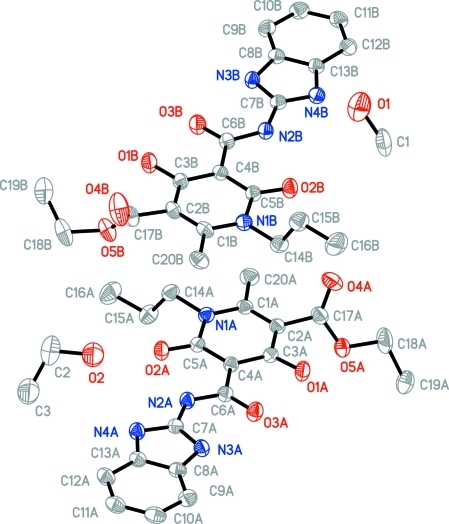
Molecular view of the title compound with atomic labelling. All atoms are shown with displacement ellipsoids drawn at the 50% probability level. Hydrogen atoms and minor components of disordered ethyl group in molecule A omitted for clarity.

**Fig. 2.**
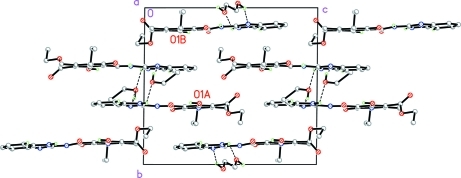
The packing of the molecules in the crystal phase. Hydrogen bonds between molecules of (I) and bridging solvate molecules are shown by dashed lines.

## supplementary crystallographic information

### Comment

*N*-acylic derivatives of 2-aminobenzoimidazole are considered as potential antithyroid drugs (Ukrainets *et al.*, 1993), for what the structures of these compounds are of particular interest. In the present paper, we report the crystal structure of ethyl-5(1*H*- benzoimidazol-2-ylcarbamoyl)-4-hydroxy-2-methyl-6-oxo-1-propyl-1,6- dihydropyridine-3-carboxylate (I). The compound exists as a ethanol:methanol solvate in a 4:2:1 ratio. The asymmetric unit contains two molecules (A and B) of I, one ethanol molecule and one methanol molecule located in a centre of symmetry. The pyridinone ring, the carbamide group and the bicyclic fragment in molecules A and B lie in a plane within 0.03 Å due to the formation of many O—H···O, N—H···O intramolecular hydrogen bonds (Table 1, top eight entries). The formation of these hydrogen bonds causes also the redistribution of the electron density within the hydroxypyridone fragment. The O2—C5, O3—C6 and C3—C4 bonds (See Supplementary Material) are elongated as compared with reported value (mean values in the literature: 1.210 Å for the C=O and 1.326 Å for the C—C bond; Bürgi & Dunitz, 1994) and the O1—C3 and C4—C5 bonds are, instead, shorter (mean values in the literature: 1.362 Å and 1.455 Å). Some elongation of the C1—C2 bond and shortening of the C2—C3 bond can be caused, probably, by the strong conjugation between endocyclic double bonds which is typical for the pyridinone ring (Low & Wilson, 1983). The substituent at the C2 atom is turned relatively to the plane of the heterocycle (C1—C2—C17—O4 torsion angle: 47.2 (4) ° in A and 59.3 (4) ° in B). The ethyl group has *sp*-conformation relatively to the C17—O4 bond (C18—O5—C17—O4 torsion angle: 6.0 (5) Å (A) 7.3 (5) Å (B)). In molecule B the ethyl group is orthogonal to the C17—O5 bond (C17—O5—C18—C19 torsion angle: 84.0 (4) °) while in the molecule A this group is disordered over three positions due to the rotation around the O5—C18 bond with populations 0.50:0.25:0.25 and C17—O5—C18—C19 torsion angle: 175.0 (5) °, 139.3 (9) %A and 92 (1) °, respectively. In the crystal phase the molecules of I form layers parallel to (010). Neighbouring layers are interlinked through H-bonds mediated by the bridging solvate molecules (Table 1, last two entries).

### Experimental

A mixture of diethyl 4-hydroxy-2-methyl-6-oxo-1-propyl-1,6-dihydro- pyridine-3,5-dicarboxylate (Ukrainets *et al.*, 2006) (3.11 g, 10.0 mmol), 2-aminobenzimidazole (1.33 g, 10.0 mmol), and DMF (1 ml) was stirred an kept on a metal bath at 160%A C for 5 min. The mixture was cooled, ethanol (30 ml) was added, the mixture was thoroughly mixed and filtered. The amino-ester obtained was washed on the filter with ethanol and dried. Yield 3.58 g (83%). m.p. 154–156%A C.

### Refinement

Crystals were multiple and poorly diffracting: in the specimen used no reflections appeared above 2θ = 50°, for what this was taken as the integration limit. Besides, some reflections were rejected in the integration process due to overlap with minor components. Restrictions on bond lengths (C*sp*^3^—C*sp*^3^ 1.54 (1) Å, C*sp*^3^—O 1.43 (1) Å) in the solvate molecules and the disordered fragment were applied during refinement. The occupation factor for the latter group were refined for a few cycles, rounded up and kept fixed for the rest of the process. Hydrogen atoms were located from electron density difference maps and further idealized, or were calculated geometrically for the disordered fragment and solvate molecules, and were refined in the riding motion approximation with *U*_iso_ constrained to be 1.5 times *U*_eq_ of the carrier atom for the methyl and hydroxyl groups and 1.2 times *U*_eq_ of the carrier atom for the other atoms.

### Crystal data

**Table:**

4C_20_H_22_N_4_O_5_·2C_2_H_6_O·CH_4_O	*Z* = 1
*M**_r_* = 1717.84	*F*(000) = 910
Triclinic, *P*1	*D*_x_ = 1.303 Mg m^−^^3^
Hall symbol: -P 1	Mo *K*α radiation, λ = 0.71073 Å
*a* = 10.5527 (4) Å	Cell parameters from 6420 reflections
*b* = 13.9720 (4) Å	θ = 2.7–32.1°
*c* = 16.0242 (5) Å	µ = 0.10 mm^−^^1^
α = 86.804 (3)°	*T* = 293 K
β = 70.980 (3)°	Plate, colourless
γ = 78.652 (3)°	0.40 × 0.30 × 0.10 mm
*V* = 2189.90 (12) Å^3^	

### Data collection

**Table:**

Oxford Diffraction Xcalibur3 diffractometer	3785 reflections with *I* > 2σ(*I*)
Radiation source: Enhance (Mo) X-ray Source	*R*_int_ = 0.030
graphite	θ_max_ = 25.0°, θ_min_ = 2.8°
Detector resolution: 16.1827 pixels mm^-1^	*h* = −12→12
ω scans	*k* = −16→16
23866 measured reflections	*l* = −19→19
7499 independent reflections	

### Refinement

**Table:**

Refinement on *F*^2^	Primary atom site location: structure-invariant direct methods
Least-squares matrix: full	Secondary atom site location: difference Fourier map
*R*[*F*^2^ > 2σ(*F*^2^)] = 0.055	Hydrogen site location: inferred from neighbouring sites
*wR*(*F*^2^) = 0.170	H-atom parameters constrained
*S* = 0.89	*w* = 1/[σ^2^(*F*_o_^2^) + (0.1051*P*)^2^] where *P* = (*F*_o_^2^ + 2*F*_c_^2^)/3
7499 reflections	(Δ/σ)_max_ = 0.001
596 parameters	Δρ_max_ = 0.44 e Å^−^^3^
9 restraints	Δρ_min_ = −0.35 e Å^−^^3^

### Special details

**Table:**

Geometry. All e.s.d.'s (except the e.s.d. in the dihedral angle between two l.s. planes) are estimated using the full covariance matrix. The cell e.s.d.'s are taken into account individually in the estimation of e.s.d.'s in distances, angles and torsion angles; correlations between e.s.d.'s in cell parameters are only used when they are defined by crystal symmetry. An approximate (isotropic) treatment of cell e.s.d.'s is used for estimating e.s.d.'s involving l.s. planes.
Refinement. Refinement of *F*^2^ against ALL reflections. The weighted *R*-factor *wR* and goodness of fit *S* are based on *F*^2^, conventional *R*-factors *R* are based on *F*, with *F* set to zero for negative *F*^2^. The threshold expression of *F*^2^ > σ(*F*^2^) is used only for calculating *R*-factors(gt) *etc*. and is not relevant to the choice of reflections for refinement. *R*-factors based on *F*^2^ are statistically about twice as large as those based on *F*, and *R*- factors based on ALL data will be even larger.

### Fractional atomic coordinates and isotropic or equivalent isotropic displacement parameters (Å^2^)

**Table:**

	*x*	*y*	*z*	*U*_iso_*/*U*_eq_	Occ. (<1)
O1	0.8136 (6)	−0.0281 (4)	0.5379 (4)	0.1256 (19)	0.50
H1	0.7474	0.0031	0.5752	0.188*	0.50
C1	0.9280 (7)	0.0168 (6)	0.5292 (5)	0.104 (3)	0.50
H13	1.0073	−0.0184	0.4859	0.156*	0.50
H12	0.9102	0.0834	0.5112	0.156*	0.50
H11	0.9436	0.0148	0.5851	0.156*	0.50
O2	0.7030 (3)	0.5258 (2)	−0.04837 (18)	0.1089 (9)	
H2	0.7304	0.5775	−0.0542	0.163*	
C2	0.6397 (5)	0.5185 (4)	−0.1135 (3)	0.1353 (17)	
H22	0.5414	0.5338	−0.0861	0.162*	
H21	0.6637	0.4517	−0.1349	0.162*	
C3	0.6811 (5)	0.5866 (4)	−0.1923 (3)	0.1401 (19)	
H33	0.6254	0.5856	−0.2291	0.210*	
H32	0.7753	0.5648	−0.2260	0.210*	
H31	0.6683	0.6519	−0.1712	0.210*	
O1A	0.93576 (19)	0.61024 (15)	0.32098 (12)	0.0634 (5)	
H1A	0.9905	0.6073	0.2709	0.095*	
O2A	0.61620 (18)	0.64723 (13)	0.16678 (12)	0.0577 (5)	
O3A	1.0200 (2)	0.62015 (14)	0.15611 (12)	0.0619 (5)	
O4A	0.6881 (3)	0.55814 (18)	0.53908 (16)	0.1086 (10)	
O5A	0.8100 (2)	0.67441 (14)	0.49024 (12)	0.0663 (6)	
N1A	0.5464 (2)	0.63870 (14)	0.31662 (13)	0.0470 (5)	
N2A	0.8745 (2)	0.62809 (14)	0.07776 (13)	0.0491 (5)	
H2A	0.7905	0.6334	0.0810	0.059*	
N3A	1.1051 (2)	0.61149 (14)	−0.02264 (14)	0.0507 (5)	
H3A	1.1476	0.6071	0.0154	0.061*	
N4A	0.9310 (2)	0.62716 (15)	−0.07671 (14)	0.0518 (6)	
C1A	0.5712 (3)	0.63431 (17)	0.39529 (16)	0.0464 (6)	
C2A	0.7019 (3)	0.62597 (17)	0.39704 (16)	0.0478 (6)	
C3A	0.8109 (3)	0.62374 (17)	0.31688 (16)	0.0454 (6)	
C4A	0.7849 (3)	0.63165 (16)	0.23636 (15)	0.0439 (6)	
C5A	0.6497 (3)	0.64032 (16)	0.23452 (17)	0.0432 (6)	
C6A	0.9007 (3)	0.62683 (17)	0.15455 (17)	0.0469 (6)	
C7A	0.9693 (3)	0.62164 (16)	−0.00579 (16)	0.0452 (6)	
C8A	1.1622 (3)	0.60966 (17)	−0.11410 (17)	0.0493 (7)	
C9A	1.2972 (3)	0.60238 (19)	−0.16880 (19)	0.0610 (8)	
H9A	1.3695	0.5956	−0.1465	0.073*	
C10A	1.3164 (3)	0.60597 (19)	−0.2586 (2)	0.0650 (8)	
H10A	1.4045	0.6019	−0.2979	0.078*	
C11A	1.2089 (3)	0.61542 (19)	−0.29163 (19)	0.0622 (8)	
H11A	1.2267	0.6175	−0.3524	0.075*	
C12A	1.0771 (3)	0.62182 (19)	−0.23733 (18)	0.0596 (7)	
H12A	1.0057	0.6276	−0.2604	0.072*	
C13A	1.0530 (3)	0.61940 (17)	−0.14619 (17)	0.0480 (6)	
C14A	0.4088 (3)	0.6390 (2)	0.31050 (19)	0.0567 (7)	
H14B	0.3559	0.6091	0.3630	0.068*	
H14A	0.4185	0.5996	0.2600	0.068*	
C15A	0.3321 (3)	0.7395 (2)	0.3014 (2)	0.0699 (8)	
H15B	0.3144	0.7773	0.3543	0.084*	
H15A	0.3880	0.7719	0.2519	0.084*	
C16A	0.1993 (3)	0.7370 (3)	0.2871 (3)	0.0963 (11)	
H16C	0.1430	0.7058	0.3363	0.144*	
H16B	0.2164	0.7010	0.2339	0.144*	
H16A	0.1533	0.8024	0.2819	0.144*	
C17A	0.7317 (3)	0.6147 (2)	0.48234 (18)	0.0608 (8)	
C18A	0.8539 (4)	0.6630 (3)	0.5673 (2)	0.0959 (12)	
H18A	0.8992	0.5962	0.5711	0.115*	0.50
H18B	0.7759	0.6785	0.6203	0.115*	0.50
H18E	0.8463	0.5990	0.5928	0.115*	0.25
H18F	0.7993	0.7123	0.6115	0.115*	0.25
H18G	0.7882	0.6362	0.6156	0.115*	0.25
H18H	0.8628	0.7256	0.5858	0.115*	0.25
C19A	0.9530 (10)	0.7334 (8)	0.5579 (7)	0.107 (4)	0.50
H19A	0.9841	0.7275	0.6083	0.161*	0.50
H19B	0.9070	0.7992	0.5542	0.161*	0.50
H19C	1.0298	0.7173	0.5053	0.161*	0.50
C19C	1.0040 (8)	0.6754 (17)	0.5334 (12)	0.098 (7)	0.25
H19G	1.0080	0.7426	0.5190	0.146*	0.25
H19H	1.0521	0.6356	0.4816	0.146*	0.25
H19I	1.0454	0.6558	0.5783	0.146*	0.25
C19D	0.9929 (14)	0.5928 (15)	0.5406 (10)	0.129 (7)	0.25
H19J	0.9796	0.5276	0.5575	0.194*	0.25
H19K	1.0502	0.6121	0.5699	0.194*	0.25
H19L	1.0357	0.5950	0.4779	0.194*	0.25
C20A	0.4504 (3)	0.6403 (2)	0.47764 (18)	0.0637 (8)	
H20C	0.3804	0.6940	0.4734	0.095*	
H20B	0.4775	0.6500	0.5277	0.095*	
H20A	0.4158	0.5807	0.4845	0.095*	
O1B	0.5138 (2)	0.15276 (16)	0.20358 (12)	0.0661 (5)	
H1B	0.4590	0.1567	0.2537	0.099*	
O2B	0.83685 (19)	0.12699 (14)	0.35437 (12)	0.0621 (5)	
O3B	0.4315 (2)	0.14907 (14)	0.36909 (11)	0.0626 (5)	
O4B	0.7561 (3)	0.0800 (2)	−0.01009 (16)	0.1132 (10)	
O5B	0.6275 (2)	0.22567 (15)	0.03465 (13)	0.0774 (6)	
N1B	0.9037 (2)	0.13316 (15)	0.20430 (13)	0.0495 (5)	
N2B	0.5847 (2)	0.12074 (14)	0.44403 (12)	0.0470 (5)	
H2B	0.6698	0.1119	0.4392	0.056*	
N3B	0.3573 (2)	0.12625 (14)	0.54523 (13)	0.0469 (5)	
H3B	0.3142	0.1335	0.5074	0.056*	
N4B	0.5335 (2)	0.10497 (15)	0.59801 (13)	0.0496 (5)	
C1B	0.8763 (3)	0.13635 (17)	0.12602 (16)	0.0501 (7)	
C2B	0.7451 (3)	0.14426 (17)	0.12605 (16)	0.0477 (6)	
C3B	0.6372 (3)	0.14685 (17)	0.20741 (16)	0.0454 (6)	
C4B	0.6659 (3)	0.13928 (16)	0.28656 (15)	0.0423 (6)	
C5B	0.8017 (3)	0.13273 (17)	0.28661 (16)	0.0449 (6)	
C6B	0.5526 (3)	0.13693 (17)	0.36914 (16)	0.0466 (6)	
C7B	0.4930 (3)	0.11702 (16)	0.52847 (16)	0.0439 (6)	
C8B	0.3012 (3)	0.12150 (17)	0.63690 (16)	0.0454 (6)	
C9B	0.1684 (3)	0.1288 (2)	0.69188 (18)	0.0568 (7)	
H9B	0.0954	0.1380	0.6701	0.068*	
C10B	0.1500 (3)	0.1216 (2)	0.78080 (19)	0.0612 (8)	
H10B	0.0620	0.1263	0.8202	0.073*	
C11B	0.2572 (3)	0.1078 (2)	0.81315 (18)	0.0589 (7)	
H11B	0.2398	0.1037	0.8739	0.071*	
C12B	0.3898 (3)	0.09979 (19)	0.75866 (17)	0.0553 (7)	
H12B	0.4622	0.0891	0.7813	0.066*	
C13B	0.4118 (3)	0.10834 (17)	0.66814 (16)	0.0446 (6)	
C14B	1.0427 (3)	0.1290 (2)	0.20801 (19)	0.0586 (7)	
H14D	1.0369	0.1665	0.2587	0.070*	
H14C	1.0953	0.1592	0.1555	0.070*	
C15B	1.1158 (3)	0.0272 (2)	0.2143 (2)	0.0689 (8)	
H15D	1.1263	−0.0096	0.1622	0.083*	
H15C	1.0619	−0.0042	0.2654	0.083*	
C16B	1.2553 (4)	0.0263 (3)	0.2224 (3)	0.1056 (13)	
H16F	1.3074	0.0599	0.1733	0.158*	
H16E	1.3020	−0.0399	0.2228	0.158*	
H16D	1.2447	0.0584	0.2764	0.158*	
C17B	0.7118 (3)	0.1451 (2)	0.04263 (18)	0.0646 (8)	
C18B	0.5741 (4)	0.2295 (3)	−0.0391 (2)	0.1005 (12)	
H18D	0.6441	0.1966	−0.0901	0.121*	
H18C	0.5483	0.2969	−0.0546	0.121*	
C19B	0.4490 (4)	0.1798 (3)	−0.0134 (3)	0.1195 (15)	
H19F	0.4750	0.1130	0.0015	0.179*	
H19E	0.4146	0.1822	−0.0622	0.179*	
H19D	0.3793	0.2132	0.0365	0.179*	
C20B	0.9947 (3)	0.1321 (2)	0.04247 (17)	0.0620 (8)	
H20F	0.9628	0.1339	−0.0074	0.093*	
H20E	1.0593	0.0727	0.0407	0.093*	
H20D	1.0379	0.1869	0.0406	0.093*	

### Atomic displacement parameters (Å^2^)

**Table:**

	*U*^11^	*U*^22^	*U*^33^	*U*^12^	*U*^13^	*U*^23^
O1	0.101 (5)	0.156 (5)	0.125 (5)	−0.011 (4)	−0.047 (4)	−0.017 (4)
C1	0.079 (5)	0.173 (8)	0.062 (5)	−0.006 (6)	−0.039 (4)	0.014 (5)
O2	0.098 (2)	0.142 (2)	0.0954 (19)	−0.0334 (19)	−0.0382 (16)	0.0141 (17)
C2	0.130 (4)	0.168 (4)	0.140 (4)	−0.030 (3)	−0.088 (4)	0.005 (4)
C3	0.123 (4)	0.218 (5)	0.106 (3)	−0.073 (4)	−0.055 (3)	0.036 (4)
O1A	0.0518 (13)	0.0949 (14)	0.0495 (11)	−0.0165 (11)	−0.0226 (9)	−0.0021 (11)
O2A	0.0486 (11)	0.0806 (12)	0.0461 (11)	−0.0094 (10)	−0.0201 (9)	0.0013 (9)
O3A	0.0469 (12)	0.0902 (14)	0.0488 (11)	−0.0154 (10)	−0.0135 (9)	−0.0049 (9)
O4A	0.165 (3)	0.1181 (19)	0.0764 (16)	−0.0805 (19)	−0.0612 (17)	0.0487 (15)
O5A	0.0851 (15)	0.0796 (13)	0.0487 (11)	−0.0282 (12)	−0.0345 (11)	0.0049 (9)
N1A	0.0458 (14)	0.0497 (12)	0.0455 (13)	−0.0111 (10)	−0.0133 (11)	0.0000 (9)
N2A	0.0455 (14)	0.0633 (13)	0.0397 (13)	−0.0116 (11)	−0.0144 (11)	0.0010 (10)
N3A	0.0497 (15)	0.0588 (13)	0.0433 (13)	−0.0082 (11)	−0.0163 (11)	0.0026 (10)
N4A	0.0543 (15)	0.0599 (13)	0.0421 (13)	−0.0104 (11)	−0.0166 (11)	−0.0010 (10)
C1A	0.0564 (18)	0.0459 (14)	0.0375 (14)	−0.0170 (13)	−0.0117 (13)	0.0006 (11)
C2A	0.0608 (19)	0.0454 (14)	0.0415 (15)	−0.0173 (13)	−0.0183 (14)	0.0031 (11)
C3A	0.0476 (17)	0.0447 (14)	0.0473 (15)	−0.0126 (12)	−0.0177 (13)	−0.0001 (11)
C4A	0.0479 (17)	0.0436 (13)	0.0410 (14)	−0.0095 (12)	−0.0149 (12)	−0.0006 (11)
C5A	0.0421 (16)	0.0444 (14)	0.0434 (15)	−0.0084 (12)	−0.0138 (13)	−0.0007 (11)
C6A	0.0456 (17)	0.0496 (14)	0.0459 (16)	−0.0090 (12)	−0.0150 (13)	−0.0013 (11)
C7A	0.0462 (17)	0.0444 (14)	0.0447 (16)	−0.0107 (12)	−0.0132 (13)	0.0017 (11)
C8A	0.0530 (18)	0.0456 (14)	0.0432 (15)	−0.0104 (13)	−0.0061 (14)	−0.0014 (11)
C9A	0.0568 (19)	0.0632 (17)	0.0613 (19)	−0.0146 (15)	−0.0148 (16)	−0.0011 (14)
C10A	0.065 (2)	0.0574 (17)	0.0575 (19)	−0.0117 (15)	−0.0001 (17)	0.0017 (14)
C11A	0.074 (2)	0.0570 (17)	0.0461 (17)	−0.0107 (16)	−0.0081 (17)	0.0026 (13)
C12A	0.074 (2)	0.0584 (16)	0.0443 (16)	−0.0114 (15)	−0.0167 (16)	0.0019 (13)
C13A	0.0531 (18)	0.0440 (14)	0.0440 (15)	−0.0084 (12)	−0.0120 (14)	0.0005 (11)
C14A	0.0465 (17)	0.0657 (17)	0.0585 (17)	−0.0180 (14)	−0.0133 (14)	−0.0005 (13)
C15A	0.058 (2)	0.075 (2)	0.079 (2)	−0.0153 (16)	−0.0236 (17)	−0.0028 (16)
C16A	0.052 (2)	0.118 (3)	0.120 (3)	−0.019 (2)	−0.029 (2)	0.000 (2)
C17A	0.074 (2)	0.0634 (17)	0.0479 (17)	−0.0168 (16)	−0.0222 (16)	0.0069 (14)
C18A	0.118 (3)	0.130 (3)	0.057 (2)	−0.022 (3)	−0.051 (2)	−0.003 (2)
C19A	0.152 (9)	0.124 (8)	0.090 (7)	−0.065 (7)	−0.076 (6)	0.003 (6)
C19C	0.135 (17)	0.103 (16)	0.107 (16)	−0.062 (14)	−0.089 (14)	0.021 (13)
C19D	0.103 (15)	0.21 (2)	0.097 (13)	−0.015 (16)	−0.075 (12)	0.031 (14)
C20A	0.065 (2)	0.0704 (18)	0.0517 (17)	−0.0201 (16)	−0.0096 (15)	−0.0001 (14)
O1B	0.0506 (13)	0.1019 (15)	0.0473 (11)	−0.0132 (11)	−0.0191 (9)	0.0058 (11)
O2B	0.0503 (12)	0.0938 (14)	0.0428 (11)	−0.0131 (10)	−0.0158 (9)	−0.0005 (10)
O3B	0.0459 (12)	0.0921 (14)	0.0451 (11)	−0.0093 (10)	−0.0110 (9)	0.0031 (9)
O4B	0.135 (2)	0.124 (2)	0.0706 (16)	0.0405 (18)	−0.0494 (16)	−0.0471 (15)
O5B	0.0963 (17)	0.0793 (14)	0.0563 (12)	0.0021 (13)	−0.0361 (12)	0.0090 (10)
N1B	0.0418 (13)	0.0587 (13)	0.0433 (13)	−0.0083 (10)	−0.0079 (10)	0.0002 (10)
N2B	0.0383 (12)	0.0632 (13)	0.0355 (12)	−0.0065 (10)	−0.0078 (10)	−0.0026 (10)
N3B	0.0404 (13)	0.0600 (13)	0.0396 (12)	−0.0076 (10)	−0.0125 (10)	−0.0022 (9)
N4B	0.0455 (14)	0.0619 (13)	0.0379 (12)	−0.0119 (11)	−0.0075 (11)	−0.0014 (10)
C1B	0.060 (2)	0.0448 (14)	0.0390 (15)	−0.0048 (13)	−0.0100 (13)	0.0027 (11)
C2B	0.0543 (18)	0.0487 (14)	0.0363 (14)	−0.0011 (13)	−0.0143 (13)	−0.0012 (11)
C3B	0.0486 (17)	0.0464 (14)	0.0410 (15)	−0.0072 (12)	−0.0149 (13)	−0.0016 (11)
C4B	0.0495 (17)	0.0408 (13)	0.0340 (13)	−0.0077 (12)	−0.0101 (12)	−0.0016 (10)
C5B	0.0411 (16)	0.0509 (15)	0.0390 (15)	−0.0064 (12)	−0.0092 (13)	0.0009 (11)
C6B	0.0485 (18)	0.0477 (14)	0.0418 (15)	−0.0068 (13)	−0.0128 (13)	−0.0038 (11)
C7B	0.0437 (16)	0.0440 (14)	0.0405 (15)	−0.0096 (12)	−0.0079 (13)	−0.0020 (11)
C8B	0.0459 (16)	0.0456 (14)	0.0405 (14)	−0.0082 (12)	−0.0083 (13)	−0.0015 (11)
C9B	0.0425 (17)	0.0750 (18)	0.0515 (17)	−0.0170 (14)	−0.0100 (14)	0.0013 (14)
C10B	0.0529 (19)	0.0715 (18)	0.0512 (18)	−0.0178 (15)	−0.0016 (15)	−0.0046 (14)
C11B	0.066 (2)	0.0674 (17)	0.0369 (15)	−0.0182 (15)	−0.0051 (15)	0.0001 (13)
C12B	0.0562 (19)	0.0634 (17)	0.0446 (16)	−0.0141 (14)	−0.0119 (14)	−0.0017 (13)
C13B	0.0489 (17)	0.0452 (14)	0.0363 (14)	−0.0100 (12)	−0.0082 (13)	−0.0024 (11)
C14B	0.0518 (17)	0.0687 (18)	0.0515 (16)	−0.0154 (15)	−0.0090 (14)	−0.0001 (13)
C15B	0.057 (2)	0.078 (2)	0.067 (2)	−0.0133 (16)	−0.0121 (16)	−0.0038 (15)
C16B	0.059 (2)	0.123 (3)	0.138 (4)	−0.021 (2)	−0.037 (2)	0.014 (3)
C17B	0.068 (2)	0.081 (2)	0.0361 (16)	−0.0040 (18)	−0.0108 (15)	−0.0032 (15)
C18B	0.126 (3)	0.118 (3)	0.062 (2)	−0.001 (2)	−0.051 (2)	0.012 (2)
C19B	0.140 (4)	0.147 (4)	0.101 (3)	−0.032 (3)	−0.077 (3)	0.013 (3)
C20B	0.062 (2)	0.0698 (18)	0.0427 (16)	−0.0084 (15)	−0.0031 (14)	0.0028 (13)

### Geometric parameters (Å, °)

**Table:**

O1—C1	1.4313 (10)	C18A—H18H	0.9700
O1—H1	0.8200	C19A—H19A	0.9600
C1—C1^i^	1.500 (14)	C19A—H19B	0.9600
C1—H13	0.9600	C19A—H19C	0.9600
C1—H12	0.9601	C19C—H19G	0.9600
C1—H11	0.9599	C19C—H19H	0.9600
O2—C2	1.428 (6)	C19C—H19I	0.9600
O2—H2	0.8200	C19D—H19J	0.9600
C2—C3	1.537 (7)	C19D—H19K	0.9600
C2—H22	0.9700	C19D—H19L	0.9600
C2—H21	0.9700	C20A—H20C	0.9600
C3—H33	0.9600	C20A—H20B	0.9600
C3—H32	0.9600	C20A—H20A	0.9600
C3—H31	0.9600	O1B—C3B	1.310 (3)
O1A—C3A	1.316 (3)	O1B—H1B	0.8200
O1A—H1A	0.8200	O2B—C5B	1.250 (3)
O2A—C5A	1.240 (3)	O3B—C6B	1.256 (3)
O3A—C6A	1.252 (3)	O4B—C17B	1.191 (3)
O4A—C17A	1.205 (3)	O5B—C17B	1.319 (4)
O5A—C17A	1.322 (3)	O5B—C18B	1.461 (4)
O5A—C18A	1.444 (3)	N1B—C1B	1.373 (3)
N1A—C1A	1.364 (3)	N1B—C5B	1.405 (3)
N1A—C5A	1.411 (3)	N1B—C14B	1.478 (3)
N1A—C14A	1.486 (3)	N2B—C6B	1.345 (3)
N2A—C6A	1.345 (3)	N2B—C7B	1.388 (3)
N2A—C7A	1.380 (3)	N2B—H2B	0.8600
N2A—H2A	0.8600	N3B—C7B	1.350 (3)
N3A—C7A	1.349 (3)	N3B—C8B	1.396 (3)
N3A—C8A	1.391 (3)	N3B—H3B	0.8600
N3A—H3A	0.8600	N4B—C7B	1.309 (3)
N4A—C7A	1.318 (3)	N4B—C13B	1.399 (3)
N4A—C13A	1.390 (3)	C1B—C2B	1.367 (4)
C1A—C2A	1.371 (4)	C1B—C20B	1.499 (4)
C1A—C20A	1.499 (4)	C2B—C3B	1.421 (3)
C2A—C3A	1.415 (4)	C2B—C17B	1.488 (4)
C2A—C17A	1.493 (4)	C3B—C4B	1.391 (3)
C3A—C4A	1.399 (3)	C4B—C5B	1.418 (4)
C4A—C5A	1.417 (3)	C4B—C6B	1.470 (3)
C4A—C6A	1.466 (4)	C8B—C9B	1.378 (4)
C8A—C13A	1.388 (4)	C8B—C13B	1.391 (4)
C8A—C9A	1.395 (4)	C9B—C10B	1.375 (4)
C9A—C10A	1.386 (4)	C9B—H9B	0.9300
C9A—H9A	0.9300	C10B—C11B	1.368 (4)
C10A—C11A	1.381 (4)	C10B—H10B	0.9300
C10A—H10A	0.9300	C11B—C12B	1.373 (4)
C11A—C12A	1.368 (4)	C11B—H11B	0.9300
C11A—H11A	0.9300	C12B—C13B	1.394 (3)
C12A—C13A	1.398 (4)	C12B—H12B	0.9300
C12A—H12A	0.9300	C14B—C15B	1.494 (4)
C14A—C15A	1.502 (4)	C14B—H14D	0.9700
C14A—H14B	0.9700	C14B—H14C	0.9700
C14A—H14A	0.9700	C15B—C16B	1.516 (4)
C15A—C16A	1.499 (4)	C15B—H15D	0.9700
C15A—H15B	0.9700	C15B—H15C	0.9700
C15A—H15A	0.9700	C16B—H16F	0.9600
C16A—H16C	0.9600	C16B—H16E	0.9600
C16A—H16B	0.9600	C16B—H16D	0.9600
C16A—H16A	0.9600	C18B—C19B	1.539 (6)
C18A—C19A	1.5390 (10)	C18B—H18D	0.9700
C18A—C19D	1.5396 (11)	C18B—H18C	0.9700
C18A—C19C	1.5396 (11)	C19B—H19F	0.9600
C18A—H18A	0.9700	C19B—H19E	0.9600
C18A—H18B	0.9700	C19B—H19D	0.9600
C18A—H18E	0.9700	C20B—H20F	0.9600
C18A—H18F	0.9700	C20B—H20E	0.9600
C18A—H18G	0.9700	C20B—H20D	0.9600
			
C1—O1—H1	107.4	H18A—C18A—H18G	76.3
O1—C1—C1^i^	126.6 (9)	H18F—C18A—H18G	68.3
O1—C1—H13	109.2	O5A—C18A—H18H	110.4
O1—C1—H12	110.2	C19D—C18A—H18H	110.4
C1^i^—C1—H12	101.3	C19C—C18A—H18H	69.3
H13—C1—H12	109.5	H18A—C18A—H18H	133.8
O1—C1—H11	109.0	H18B—C18A—H18H	76.6
C1^i^—C1—H11	99.1	H18E—C18A—H18H	137.0
H13—C1—H11	109.5	H18G—C18A—H18H	108.6
H12—C1—H11	109.5	C18A—C19A—H19A	109.5
C2—O2—H2	109.5	C18A—C19A—H19B	109.5
O2—C2—C3	113.5 (3)	H19A—C19A—H19B	109.5
O2—C2—H22	108.9	C18A—C19A—H19C	109.5
C3—C2—H22	108.9	H19A—C19A—H19C	109.5
O2—C2—H21	108.9	H19B—C19A—H19C	109.5
C3—C2—H21	108.9	C18A—C19C—H19G	109.5
H22—C2—H21	107.7	C18A—C19C—H19H	109.5
C2—C3—H33	109.5	H19G—C19C—H19H	109.5
C2—C3—H32	109.5	C18A—C19C—H19I	109.5
H33—C3—H32	109.5	H19G—C19C—H19I	109.5
C2—C3—H31	109.5	H19H—C19C—H19I	109.5
H33—C3—H31	109.5	C18A—C19D—H19J	109.5
H32—C3—H31	109.5	C18A—C19D—H19K	109.5
C3A—O1A—H1A	109.5	H19J—C19D—H19K	109.5
C17A—O5A—C18A	116.9 (2)	C18A—C19D—H19L	109.5
C1A—N1A—C5A	122.9 (2)	H19J—C19D—H19L	109.5
C1A—N1A—C14A	122.5 (2)	H19K—C19D—H19L	109.5
C5A—N1A—C14A	114.6 (2)	C1A—C20A—H20C	109.5
C6A—N2A—C7A	126.5 (2)	C1A—C20A—H20B	109.5
C6A—N2A—H2A	116.8	H20C—C20A—H20B	109.5
C7A—N2A—H2A	116.8	C1A—C20A—H20A	109.5
C7A—N3A—C8A	105.9 (2)	H20C—C20A—H20A	109.5
C7A—N3A—H3A	127.1	H20B—C20A—H20A	109.5
C8A—N3A—H3A	127.1	C3B—O1B—H1B	109.5
C7A—N4A—C13A	103.8 (2)	C17B—O5B—C18B	116.8 (2)
N1A—C1A—C2A	120.2 (2)	C1B—N1B—C5B	122.4 (2)
N1A—C1A—C20A	117.2 (2)	C1B—N1B—C14B	122.4 (2)
C2A—C1A—C20A	122.6 (2)	C5B—N1B—C14B	115.2 (2)
C1A—C2A—C3A	119.7 (2)	C6B—N2B—C7B	126.0 (2)
C1A—C2A—C17A	120.8 (2)	C6B—N2B—H2B	117.0
C3A—C2A—C17A	119.4 (3)	C7B—N2B—H2B	117.0
O1A—C3A—C4A	122.0 (2)	C7B—N3B—C8B	105.7 (2)
O1A—C3A—C2A	118.0 (2)	C7B—N3B—H3B	127.2
C4A—C3A—C2A	120.0 (2)	C8B—N3B—H3B	127.2
C3A—C4A—C5A	120.3 (2)	C7B—N4B—C13B	103.4 (2)
C3A—C4A—C6A	118.5 (2)	C2B—C1B—N1B	120.2 (2)
C5A—C4A—C6A	121.2 (2)	C2B—C1B—C20B	122.4 (2)
O2A—C5A—N1A	118.0 (2)	N1B—C1B—C20B	117.4 (3)
O2A—C5A—C4A	125.2 (2)	C1B—C2B—C3B	119.8 (2)
N1A—C5A—C4A	116.8 (2)	C1B—C2B—C17B	121.8 (2)
O3A—C6A—N2A	121.2 (2)	C3B—C2B—C17B	118.3 (3)
O3A—C6A—C4A	121.2 (2)	O1B—C3B—C4B	122.8 (2)
N2A—C6A—C4A	117.6 (2)	O1B—C3B—C2B	117.3 (2)
N4A—C7A—N3A	114.5 (2)	C4B—C3B—C2B	119.8 (2)
N4A—C7A—N2A	121.1 (2)	C3B—C4B—C5B	120.3 (2)
N3A—C7A—N2A	124.4 (2)	C3B—C4B—C6B	118.3 (2)
C13A—C8A—N3A	105.5 (2)	C5B—C4B—C6B	121.4 (2)
C13A—C8A—C9A	123.1 (2)	O2B—C5B—N1B	117.9 (2)
N3A—C8A—C9A	131.4 (3)	O2B—C5B—C4B	124.8 (2)
C10A—C9A—C8A	115.6 (3)	N1B—C5B—C4B	117.3 (2)
C10A—C9A—H9A	122.2	O3B—C6B—N2B	121.9 (2)
C8A—C9A—H9A	122.2	O3B—C6B—C4B	121.0 (2)
C11A—C10A—C9A	122.1 (3)	N2B—C6B—C4B	117.1 (2)
C11A—C10A—H10A	119.0	N4B—C7B—N3B	115.3 (2)
C9A—C10A—H10A	119.0	N4B—C7B—N2B	121.6 (2)
C12A—C11A—C10A	121.8 (3)	N3B—C7B—N2B	123.2 (2)
C12A—C11A—H11A	119.1	C9B—C8B—C13B	122.8 (2)
C10A—C11A—H11A	119.1	C9B—C8B—N3B	132.0 (2)
C11A—C12A—C13A	118.0 (3)	C13B—C8B—N3B	105.2 (2)
C11A—C12A—H12A	121.0	C10B—C9B—C8B	116.2 (3)
C13A—C12A—H12A	121.0	C10B—C9B—H9B	121.9
C8A—C13A—N4A	110.3 (2)	C8B—C9B—H9B	121.9
C8A—C13A—C12A	119.5 (3)	C11B—C10B—C9B	122.1 (3)
N4A—C13A—C12A	130.2 (3)	C11B—C10B—H10B	119.0
N1A—C14A—C15A	113.3 (2)	C9B—C10B—H10B	119.0
N1A—C14A—H14B	108.9	C10B—C11B—C12B	122.0 (3)
C15A—C14A—H14B	108.9	C10B—C11B—H11B	119.0
N1A—C14A—H14A	108.9	C12B—C11B—H11B	119.0
C15A—C14A—H14A	108.9	C11B—C12B—C13B	117.4 (3)
H14B—C14A—H14A	107.7	C11B—C12B—H12B	121.3
C16A—C15A—C14A	112.1 (3)	C13B—C12B—H12B	121.3
C16A—C15A—H15B	109.2	C8B—C13B—C12B	119.5 (2)
C14A—C15A—H15B	109.2	C8B—C13B—N4B	110.5 (2)
C16A—C15A—H15A	109.2	C12B—C13B—N4B	130.1 (2)
C14A—C15A—H15A	109.2	N1B—C14B—C15B	112.9 (2)
H15B—C15A—H15A	107.9	N1B—C14B—H14D	109.0
C15A—C16A—H16C	109.5	C15B—C14B—H14D	109.0
C15A—C16A—H16B	109.5	N1B—C14B—H14C	109.0
H16C—C16A—H16B	109.5	C15B—C14B—H14C	109.0
C15A—C16A—H16A	109.5	H14D—C14B—H14C	107.8
H16C—C16A—H16A	109.5	C14B—C15B—C16B	111.3 (3)
H16B—C16A—H16A	109.5	C14B—C15B—H15D	109.4
O4A—C17A—O5A	122.9 (3)	C16B—C15B—H15D	109.4
O4A—C17A—C2A	124.1 (3)	C14B—C15B—H15C	109.4
O5A—C17A—C2A	113.0 (2)	C16B—C15B—H15C	109.4
O5A—C18A—C19A	107.5 (5)	H15D—C15B—H15C	108.0
O5A—C18A—C19D	106.6 (6)	C15B—C16B—H16F	109.5
C19A—C18A—C19D	78.2 (10)	C15B—C16B—H16E	109.5
O5A—C18A—C19C	104.9 (8)	H16F—C16B—H16E	109.5
C19D—C18A—C19C	45.0 (10)	C15B—C16B—H16D	109.5
O5A—C18A—H18A	110.2	H16F—C16B—H16D	109.5
C19A—C18A—H18A	110.2	H16E—C16B—H16D	109.5
C19C—C18A—H18A	79.8	O4B—C17B—O5B	124.5 (3)
O5A—C18A—H18B	110.2	O4B—C17B—C2B	123.6 (3)
C19A—C18A—H18B	110.2	O5B—C17B—C2B	112.0 (2)
C19D—C18A—H18B	137.0	O5B—C18B—C19B	109.9 (3)
C19C—C18A—H18B	137.6	O5B—C18B—H18D	109.7
H18A—C18A—H18B	108.5	C19B—C18B—H18D	109.7
O5A—C18A—H18E	110.8	O5B—C18B—H18C	109.7
C19A—C18A—H18E	134.7	C19B—C18B—H18C	109.7
C19D—C18A—H18E	68.4	H18D—C18B—H18C	108.2
C19C—C18A—H18E	110.8	C18B—C19B—H19F	109.5
H18B—C18A—H18E	78.2	C18B—C19B—H19E	109.5
O5A—C18A—H18F	110.8	H19F—C19B—H19E	109.5
C19A—C18A—H18F	78.4	C18B—C19B—H19D	109.5
C19D—C18A—H18F	140.3	H19F—C19B—H19D	109.5
C19C—C18A—H18F	110.8	H19E—C19B—H19D	109.5
H18A—C18A—H18F	132.8	C1B—C20B—H20F	109.5
H18E—C18A—H18F	108.8	C1B—C20B—H20E	109.5
O5A—C18A—H18G	110.4	H20F—C20B—H20E	109.5
C19A—C18A—H18G	136.2	C1B—C20B—H20D	109.5
C19D—C18A—H18G	110.4	H20F—C20B—H20D	109.5
C19C—C18A—H18G	142.4	H20E—C20B—H20D	109.5
			
C5A—N1A—C1A—C2A	−3.5 (3)	C17A—O5A—C18A—C19C	139.4 (9)
C14A—N1A—C1A—C2A	174.5 (2)	C5B—N1B—C1B—C2B	3.3 (3)
C5A—N1A—C1A—C20A	175.7 (2)	C14B—N1B—C1B—C2B	−177.2 (2)
C14A—N1A—C1A—C20A	−6.2 (3)	C5B—N1B—C1B—C20B	−177.4 (2)
N1A—C1A—C2A—C3A	1.2 (3)	C14B—N1B—C1B—C20B	2.0 (3)
C20A—C1A—C2A—C3A	−178.0 (2)	N1B—C1B—C2B—C3B	−1.5 (4)
N1A—C1A—C2A—C17A	−176.1 (2)	C20B—C1B—C2B—C3B	179.3 (2)
C20A—C1A—C2A—C17A	4.7 (4)	N1B—C1B—C2B—C17B	−178.2 (2)
C1A—C2A—C3A—O1A	−176.3 (2)	C20B—C1B—C2B—C17B	2.6 (4)
C17A—C2A—C3A—O1A	1.0 (3)	C1B—C2B—C3B—O1B	−178.8 (2)
C1A—C2A—C3A—C4A	1.1 (3)	C17B—C2B—C3B—O1B	−2.0 (4)
C17A—C2A—C3A—C4A	178.3 (2)	C1B—C2B—C3B—C4B	−1.3 (4)
O1A—C3A—C4A—C5A	176.2 (2)	C17B—C2B—C3B—C4B	175.5 (2)
C2A—C3A—C4A—C5A	−1.0 (3)	O1B—C3B—C4B—C5B	179.8 (2)
O1A—C3A—C4A—C6A	−1.4 (3)	C2B—C3B—C4B—C5B	2.4 (3)
C2A—C3A—C4A—C6A	−178.6 (2)	O1B—C3B—C4B—C6B	0.6 (3)
C1A—N1A—C5A—O2A	−177.9 (2)	C2B—C3B—C4B—C6B	−176.8 (2)
C14A—N1A—C5A—O2A	3.9 (3)	C1B—N1B—C5B—O2B	178.1 (2)
C1A—N1A—C5A—C4A	3.4 (3)	C14B—N1B—C5B—O2B	−1.4 (3)
C14A—N1A—C5A—C4A	−174.7 (2)	C1B—N1B—C5B—C4B	−2.2 (3)
C3A—C4A—C5A—O2A	−179.6 (2)	C14B—N1B—C5B—C4B	178.3 (2)
C6A—C4A—C5A—O2A	−2.1 (4)	C3B—C4B—C5B—O2B	179.0 (2)
C3A—C4A—C5A—N1A	−1.1 (3)	C6B—C4B—C5B—O2B	−1.8 (4)
C6A—C4A—C5A—N1A	176.44 (19)	C3B—C4B—C5B—N1B	−0.7 (3)
C7A—N2A—C6A—O3A	0.7 (4)	C6B—C4B—C5B—N1B	178.46 (19)
C7A—N2A—C6A—C4A	−178.2 (2)	C7B—N2B—C6B—O3B	−1.2 (4)
C3A—C4A—C6A—O3A	−3.1 (3)	C7B—N2B—C6B—C4B	179.1 (2)
C5A—C4A—C6A—O3A	179.3 (2)	C3B—C4B—C6B—O3B	−5.5 (3)
C3A—C4A—C6A—N2A	175.7 (2)	C5B—C4B—C6B—O3B	175.4 (2)
C5A—C4A—C6A—N2A	−1.9 (3)	C3B—C4B—C6B—N2B	174.2 (2)
C13A—N4A—C7A—N3A	0.3 (3)	C5B—C4B—C6B—N2B	−5.0 (3)
C13A—N4A—C7A—N2A	179.0 (2)	C13B—N4B—C7B—N3B	−1.2 (3)
C8A—N3A—C7A—N4A	−0.3 (3)	C13B—N4B—C7B—N2B	178.3 (2)
C8A—N3A—C7A—N2A	−179.0 (2)	C8B—N3B—C7B—N4B	1.2 (3)
C6A—N2A—C7A—N4A	−177.6 (2)	C8B—N3B—C7B—N2B	−178.3 (2)
C6A—N2A—C7A—N3A	1.0 (4)	C6B—N2B—C7B—N4B	−177.0 (2)
C7A—N3A—C8A—C13A	0.2 (2)	C6B—N2B—C7B—N3B	2.4 (4)
C7A—N3A—C8A—C9A	178.7 (3)	C7B—N3B—C8B—C9B	178.3 (3)
C13A—C8A—C9A—C10A	0.3 (4)	C7B—N3B—C8B—C13B	−0.7 (2)
N3A—C8A—C9A—C10A	−178.0 (2)	C13B—C8B—C9B—C10B	−0.4 (4)
C8A—C9A—C10A—C11A	−0.5 (4)	N3B—C8B—C9B—C10B	−179.3 (2)
C9A—C10A—C11A—C12A	0.0 (4)	C8B—C9B—C10B—C11B	−0.2 (4)
C10A—C11A—C12A—C13A	0.6 (4)	C9B—C10B—C11B—C12B	−0.3 (4)
N3A—C8A—C13A—N4A	0.0 (3)	C10B—C11B—C12B—C13B	1.3 (4)
C9A—C8A—C13A—N4A	−178.7 (2)	C9B—C8B—C13B—C12B	1.5 (4)
N3A—C8A—C13A—C12A	178.9 (2)	N3B—C8B—C13B—C12B	−179.4 (2)
C9A—C8A—C13A—C12A	0.2 (4)	C9B—C8B—C13B—N4B	−179.1 (2)
C7A—N4A—C13A—C8A	−0.2 (3)	N3B—C8B—C13B—N4B	0.0 (3)
C7A—N4A—C13A—C12A	−179.0 (2)	C11B—C12B—C13B—C8B	−1.9 (4)
C11A—C12A—C13A—C8A	−0.7 (4)	C11B—C12B—C13B—N4B	178.8 (2)
C11A—C12A—C13A—N4A	178.0 (2)	C7B—N4B—C13B—C8B	0.7 (3)
C1A—N1A—C14A—C15A	97.7 (3)	C7B—N4B—C13B—C12B	180.0 (2)
C5A—N1A—C14A—C15A	−84.2 (3)	C1B—N1B—C14B—C15B	−94.4 (3)
N1A—C14A—C15A—C16A	174.8 (3)	C5B—N1B—C14B—C15B	85.0 (3)
C18A—O5A—C17A—O4A	6.0 (5)	N1B—C14B—C15B—C16B	−177.3 (3)
C18A—O5A—C17A—C2A	−174.9 (3)	C18B—O5B—C17B—O4B	7.1 (5)
C1A—C2A—C17A—O4A	47.2 (4)	C18B—O5B—C17B—C2B	−172.5 (3)
C3A—C2A—C17A—O4A	−130.0 (3)	C1B—C2B—C17B—O4B	59.4 (4)
C1A—C2A—C17A—O5A	−131.9 (3)	C3B—C2B—C17B—O4B	−117.4 (4)
C3A—C2A—C17A—O5A	50.9 (3)	C1B—C2B—C17B—O5B	−121.0 (3)
C17A—O5A—C18A—C19A	175.1 (5)	C3B—C2B—C17B—O5B	62.3 (3)
C17A—O5A—C18A—C19D	92.5 (11)	C17B—O5B—C18B—C19B	84.1 (4)

Symmetry codes: (i) −*x*+2, −*y*, −*z*+1.

### Hydrogen-bond geometry (Å, °)

**Table:**

*D*—H···*A*	*D*—H	H···*A*	*D*···*A*	*D*—H···*A*
O1A—H1A···O3A	0.82	1.77	2.503 (2)	149
O1B—H1B···O3B	0.82	1.78	2.508 (2)	148
N2A—H2A···O2A	0.86	1.88	2.600 (3)	140
N2B—H2B···O2B	0.86	1.88	2.596 (3)	139
N3A—H3A···O2^ii^	0.86	2.34	2.968 (3)	130
N3A—H3A···O3A	0.86	2.21	2.710 (3)	117
N3B—H3B···O1^iii^	0.86	2.46	3.106 (6)	132
N3B—H3B···O3B	0.86	2.18	2.691 (3)	118
O2—H2···N4A	0.82	2.27	2.926 (3)	138
O1—H1···N4B	0.82	2.35	3.050 (6)	144

Symmetry codes: (ii) −*x*+2, −*y*+1, −*z*; (iii) −*x*+1, −*y*, −*z*+1.
